# Endovascular therapies for hepatic artery stenosis post liver transplantation

**DOI:** 10.1186/s42155-022-00338-7

**Published:** 2022-12-08

**Authors:** I. Khati, A. Jacquier, F. Cadour, A. Bartoli, M. Graber, J. Hardwigsen, F. Tradi, P.-A. Barral

**Affiliations:** 1 Department of Radiology, CHU Timone 2, Marseille APHM, Marseille, France; 2 Department of Surgery, CHU Timone 2, Marseille APHM, Marseille, France

**Keywords:** Stent, Hepatic artery, Liver transplant, Angioplasty, Stenosis

## Abstract

**Purpose:**

To evaluate primary patency at 12 months after endovascular therapies in hepatic artery stenosis.

**Methods:**

A retrospective review of all endovascular interventions for hepatic artery stenosis (HAS) after liver transplantation that occurred between June 2013 and November 2020 was performed at a single institution in France. Follow up occurred from 1 month to 4 years (median 15 months). The treatment consisted of dilation with a balloon or stent. We analyzed short-term (technical success and complications) and long-term outcomes (liver function, arterial patency, graft survival at 12 months (GS), and reintervention). We also compared percutaneous balloon angioplasty (PBA) with stent placement. PBA alone was used if < 30% residual stenosis of the hepatic artery was achieved. Stenting was performed if there was greater than 30% residual stenosis and in the case of complications (dissection or rupture).

**Results:**

A total of 18 stenoses were suspected on the basis of routine surveillance duplex ultrasound imaging (peak systolic velocity > 200 cm/s, systolic accelerating time > 10 ms and resistive index < 0.5), all of which were confirmed by angio CT, but only 17 were confirmed by angiography.

Seventeen patients were included (14 males, mean age 57 years; and three females, mean age 58 years).

Interventions were performed in 17 cases (95%) with PBA only (5/17), stent only (5/17) or both (4/17).

Immediate technical success was 100%. Major complications occurred in 1 of 17 cases (5.8%), consisting of target vessel dissection. The analysis of the three (groups PBA only, stent only or both) showed the same procedural success (100%), GS (100%) and normal liver function after the procedures but different rates of complications (20% vs. 0% vs. 0%), arterial patency at 12 months (60% vs. 80% vs. 85%) (*p* = 0.4), early stenosis (40% vs. 80% vs. 0%) or late stenosis (60% vs. 20% vs. 100%) and requirement for reintervention (40% vs. 20% vs. 14%) (*p* = 0.56).

**Conclusion:**

This study suggests that PBA, stent, or both procedures show the same primary patency at 12 months.

It is probably not a definitive answer, but these treatments are safe and effective for extending graft survival in the context of graft shortages.

## Introduction

The prevalence of hepatic artery stenosis (HAS) after liver transplantation is reported between 2 and 13% (Wozney et al., 1986; Abbasoglu et al., 1997; Kodama et al., 2006; Hamby et al., 2013; Mondragon et al., 1994; Dodd et al., 1994). It can lead to major complication, and be responsible for high rates of morbidity and mortality (Wozney et al., 1986; Orons et al., 1995a; Chen et al., 2009).

HAS is also a major risk factor for significant hepatic artery thrombosis (HAT), which is a formal indication for retransplantation (Mourad et al., 2014; Goldsmith et al., 1489). For these reasons, the early diagnosis and treatment of HAS by endovascular therapies is critical to avoid HAT and preserve transplant liver function .

Endovascular management of arterial steno-occlusive disease has emerged as a less invasive alternative to surgical intervention in recent years (Wozney et al., 1986; Rostambeigi et al., 2013). The technique involves percutaneous balloon angioplasty (PBA), stent placement or both. Few studies have shown the superiority of one technique over the other.

The purpose of our study was to evaluate primary patency at 12 months after endovascular therapies in hepatic artery stenosis and compare PBA alone with stenting alone or both PBA and stenting. The secondary outcomes were assisted patency at 12 months, liver function after the procedure and prognosis between early and late stenosis.

## Material and methods

A retrospective review of all endovascular interventions for HAS after liver transplantation that occurred between June 2013 and November 2020 was performed at a single institution that performs 70–80 liver transplants per year.

The patients were selected utilizing a procedural database (X-plore) using the different keywords (stenting, hepatic artery, transplant, liver, PBA).

Approval for this retrospective review study was obtained from the institutional review board.

To provide the optimal treatment for the patients, we used a score that combines the following criteria (Kodama et al., 2006; Hamby et al., 2013):

- Us: resistive index < 0.5, systolic accelerating time > 10 ms, peak systolic velocity > 200 cm/s
- CT, MR or angiography

**Fig. 1:**
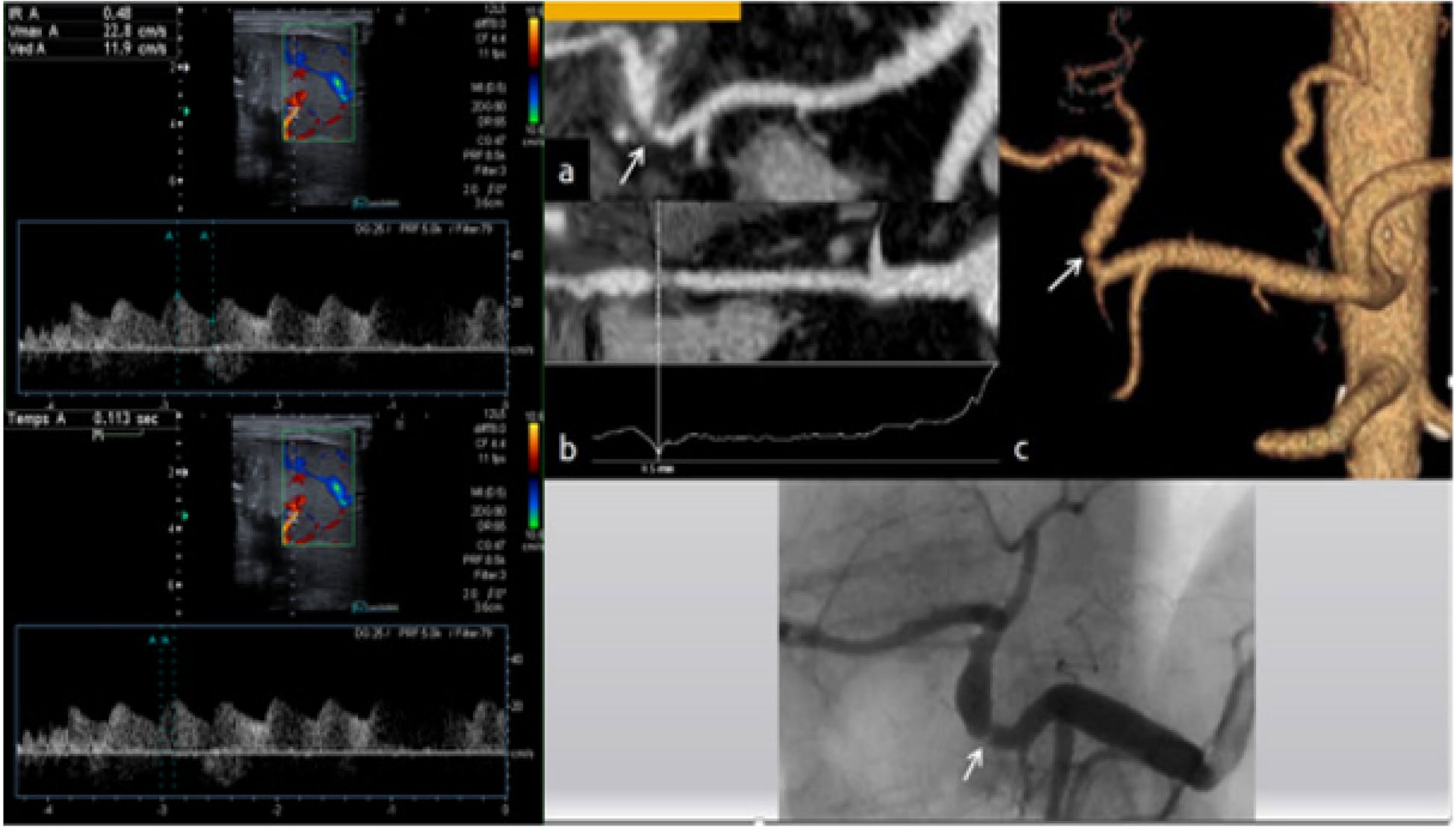
Typical tardus parvus (IR = 0,48, TAS =0,11 s) and confirmation of a significant arterial stenosis with angio CT and angiography

A significant artery stenosis was suspected when typical tardus parvus pattern, defined by a RI < 0.5 and SAT > 10 ms, was identified in the hepatic parenchyma (left, right lobe or both) associated or not with a PSV > 200 cm/s Fig. 1.

During the follow-up, all patients had Us exploration (every 3 months). When a significant stenosis was suspected a CT or MRI was systematically realized to confirm or unconfirm the stenosis.

Hepatic artery stenosis was defined as focal diameter narrowing of the hepatic artery measuring > 70% by visual estimate (Sabri et al., 2011).

Initial technical success was defined as < 30% residual stenosis of the treated hepatic artery by visual estimation of the final arteriogram (Hamby et al., 2013). Neither intravascular ultrasound imaging nor pressure gradients were used in this series. Follow up occurred from 1 month to 4 years (median 15 months).

Early stenosis was defined as a stenosis appearing within 30 days after transplantation and late stenosis after 30 days.

Primary patency was defined as the duration of patency without revision, and primary assisted patency was defined as the duration of patency after successful revision.

Endovascular reintervention was define by the recurrence of a significant stenosis during the follow up and the necessity of restenting or doing a new percutaneous angioplasty.

The decision to intervene was based exclusively on imaging studies after multidisciplinary consultation meeting.

PBA alone was used if < 30% residual stenosis of the hepatic artery was achieved. Stenting was performed if there was greater than 30% residual stenosis and in the case of complications (dissection or rupture) (Hamby et al., 2013).

### Endovascular therapies and technique

The treatment consisted of dilating with balloon or stent. We analyzed short-term (technical success and complications) and long-term outcomes (liver function, arterial patency, graft survival at 12 months (GS), and reintervention). We also compared percutaneous balloon angioplasty (PBA) with stent placement or both procedures.

In brief, A 6-F introducer sheath was placed in the common femoral artery. A 6F renal double curve catheter (RDC, Boston Scientific) and a 0.035 guide wire (Terumo Medical Corp.) were used to select the celiac trunk or superior mesenteric artery. Then, the guide wire was placed in the gastroduodenal artery or distal common hepatic artery, and a 5F Cobra catheter (Terumo Medical Corp.) was advanced for more support. A 0.014-in. or 0.0016-in. guide wire was used to cross the lesion with or without a microcatheter, depending on the difficulty of crossing the stenosis. All patients received therapeutic weight-based bolus heparin (50 u/kg) at the beginning of the procedure. Subsequent stenting was selectively performed when stent placement was technically feasible and in cases of > 30% residual stenosis after angioplasty. Balloon-expandable (Palmas Blue, Cordis, Switzerland) or rebel (Boston Scientific, Marlborough, Massachusetts) stents were used. Initial balloon (ussv, boston scientific) sizing was conservative (4-5 mm), generally choosing a diameter thought to be at least 1 mm smaller than the reference vessel. Larger balloons were then used as needed to match the reference vessel. Stent diameters were also chosen to match the reference vessel without oversizing.

For our study to be reproductible, we used the CIRSE classification to grade the complications (Filippiadis et al., 2017).

Ultrasound imaging was performed on all treated patients at 1–7 days, 3 months, 6 months, 12 months and yearly thereafter, if no stenosis was detected. More frequent follow up was conducted if a small recurrent stenosis was detected.

We defined three groups of patients: group1 (patients treated only by PBA), group 2 (patients treated only by stenting precessed or not by PBA) and group 3 (patients treated by stenting and PBA after stenting).

All patients received dual antiplatelets: group 1 (with PBA only) received long-term acetylsalicylic acid and clopidogrel for 6 weeks. Groups 2 and 3 (with stents) received lifelong acetylsalicylic acid and clopidogrel for 1 year.

### Statistical analysis

Chart review was used to determine graft survival and identify additional interventions or complications during the follow up period. Primary, primary-assisted patency, and graft survival rates were recorded and analyzed using survival analysis using the Kaplan-Meier method.

The Chi^2^ test was used for comparisons among the three groups. A *p* value < 0.05 was considered statistically significant. The statistical analysis was performed using SPSS software (version 24; SPSS, Chicago, IL).

## Results

Four hundred thirteen liver transplants were performed, with 4% (17/413) significant HAS detected, and treatment with PBA (29%; 5/17), stent (29%; 5/17) or both (42%; 7/17) was conducted. All patients underwent orthotopic transplantation. Among the grafts, 150 came from living donors, the others from cadaveric donors. For 20 patients it was the second liver transplant (most often due to recurrence of the initial disease on the graft).

A total of 18 stenoses were suspected in 18 patients by routine surveillance duplex ultrasound imaging (peak systolic velocity > 200 cm/s, systolic acceleration time (SAT) > 10 ms and resistive index (RI) < 0.5), all of which were confirmed by angio CT, but only 17 were confirmed by angiography. One patient was finally excluded because it was linked to plicature.

The medium delay between graft and endovascular treatment was 178.5 days (6 to 1000 days).

Twenty-one interventions for HAS were performed in 17 patients (14 male; mean age 57 years and three females; mean age 58 years) during the survey. No one required revision surgery or liver retransplantation.

The immediate technical success was 100%.

**Fig. 2:**
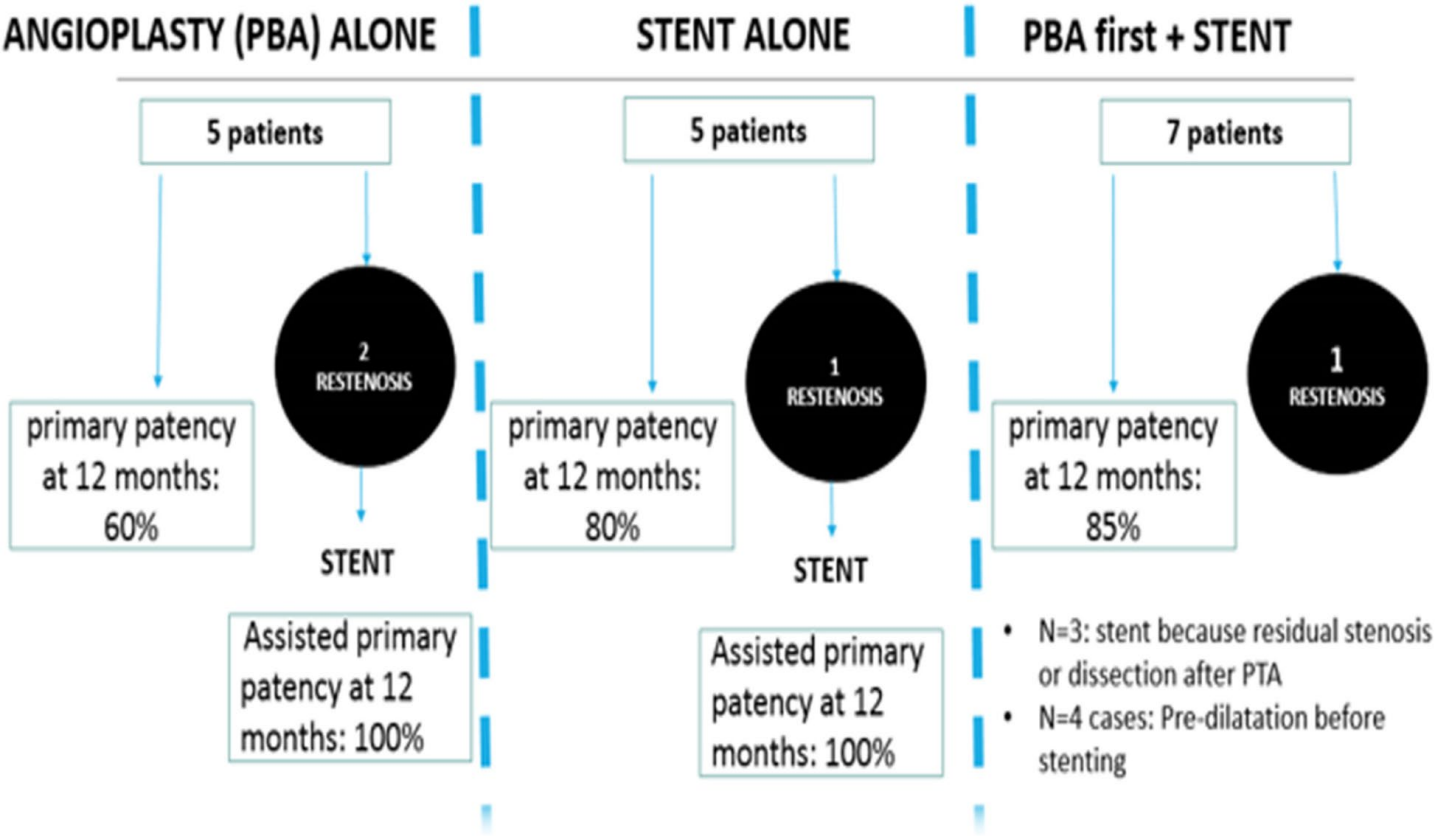
General plan of the study

**Fig. 3:**
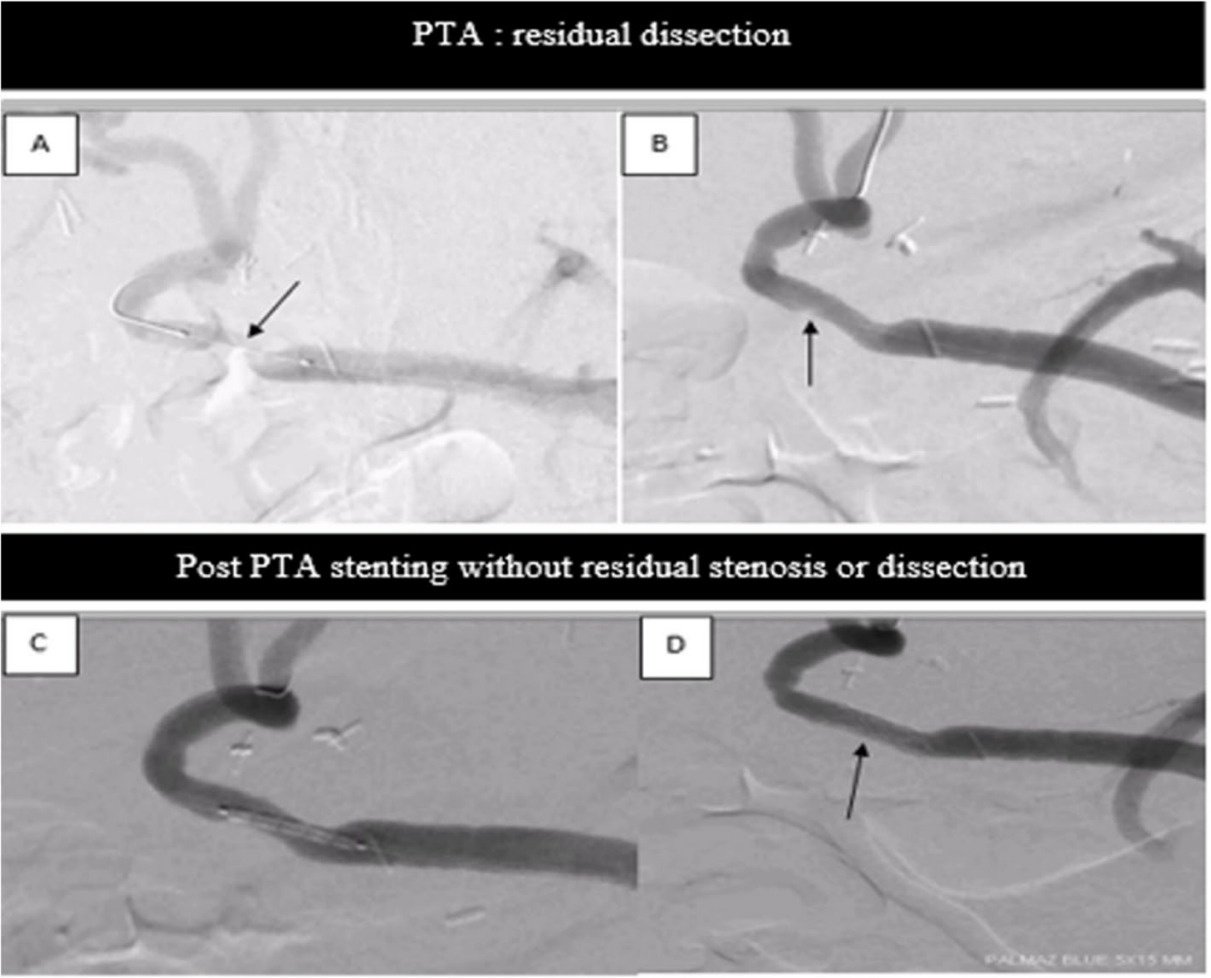
**A** Digital subtraction angiogram (DSA) of the hepatic artery shows a focal HAS at the anastomosis (arrow). **B** Post balloon angioplasty (PBA), DSA shows optimal results with residual stenosis < 30% but dissection (arrow). **C** & **D** show the deployment of a covered stent with a good primary result

There were four reinterventions in four patients for recurrent HAS: 50% (2/4) in group 1 with PBA only, and 25% each (1/4) in group 2 with stent only and in group 3 with PBA and stenting (Figs. 2 and 3).

Primary patency rates at 6, 12 and 20 months with Kaplan-Meier analysis were 80%, 60%, and 40%, respectively, in group 1 (PBA only) vs 100%, 80%, and 60% in group 2 (stenting only) vs 100%, 85%, and 60% in group 3 (PBA and stenting). (*p* = 0.4) (Fig. 2).

The primary patency rates for all patients (all groups combined) at 6, 12 and 20 months were 90%, 76%, and 55%, respectively (Fig. 2).

The assisted patency rate at 12 months was 100% (Fig. 2).

The graft survival rate at 12 months was 100%.

**Fig. 4:**
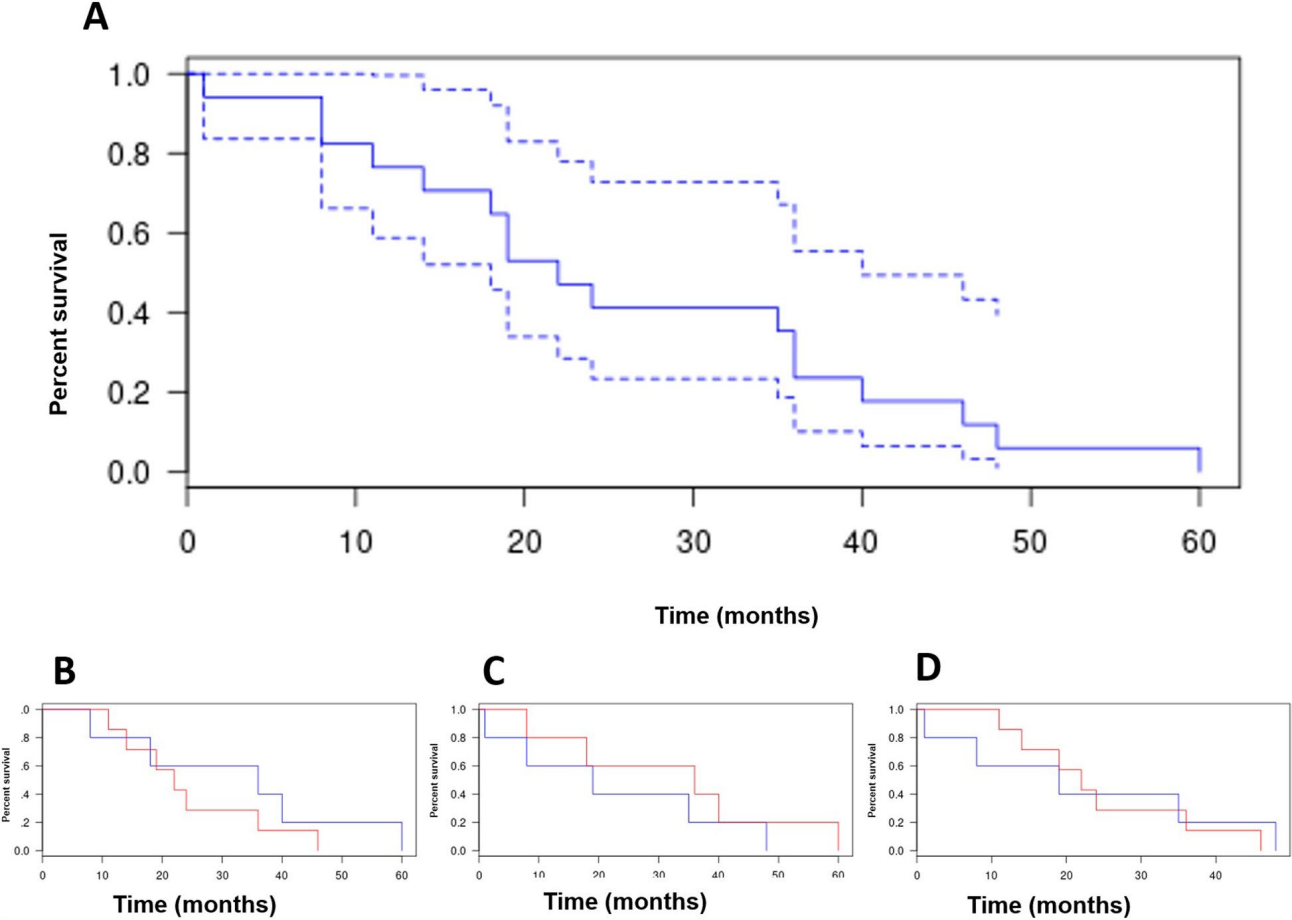
Kaplan-Meier curves log analysis showing primary patency in the total cohort (**A**) and comparing primary patency between PBA and stenting (**B**, **C**, **D**). **B** comparison between the group 1 (blue curve) and the group 3 (red curve); **C** comparison between the group 2 (red curve) and the group 3 (blue curve); **D** comparison between group 1 (blue curve) and group 2 (red curve)

Major complications occurred in 1 of 17 cases (5.8%) in group 1 (1/5; 20%), consisting of target vessel dissection (grade 3 of the CIRSE classification), which was treated with a covered stent (Fig. 4).

The rate of mortality during follow-up was 0%, and no mortality appeared to be associated with the endoluminal intervention itself.

Normal liver function at 12 months after the procedure was observed in all patients.

Early stenosis (< 30 days after graft) was observed in 41% of the population (7/17). The late stenosis rate was 86% (6/7) in group 3 vs 40% (4/10) in groups 1 and 2 (*p* = 0.056).

The pooled estimates for endovascular reintervention (PBA or restenting) showed a trend but no significant difference (reintervention in 30% (3/10) of patients in group 1 and 2 vs 14% (1/7) of patients in group 3 (*p* = 0.054)).

## Discussion

Stenosis has been found to occur at the anastomotic site in 70% of cases (Kodama et al., 2006) and within 3 months after transplantation (Wozney et al., 1986; Abbasoglu et al., 1997; Kodama et al., 2006; Mondragon et al., 1994; Dodd et al., 1994; Glockner et al., 2000).

Stenosis of the hepatic artery causes thrombosis because the hepatic arterial flow is static, resulting in hepatic necrosis (Wozney et al., 1986; Abbasoglu et al., 1997; Orons et al., 1995b; Cotroneo et al., 2002). Ischemia of the bile duct is also caused by hepatic artery stenosis, as the bile duct is fed primarily by the hepatic artery.

Our study suggests that PBA and stenting are similarly effective and safe in the treatment of HAS after liver transplantation. This might be contrary to the idea that stenting may be superior to PBA, but the results were consistent in terms of all short- and long-term outcomes of procedural success, complications, long-term patency, and graft and patient survival. Moreover, reinterventions were similarly in the three groups of patients.

Huang et al. (Huang et al., 2006) suggests that PBA is superior to stent placement; Saad et al. (Saad et al., 2005) suggests the same efficiency of these treatments. In contrast, Ueno et al. (Ueno et al., 2006) reported 26 patients with stent placement who had fewer complications and shorter length of hospital stay compared to PBA, although restenosis was seen more often in the stent group.

However, PBA seems to offer safe results as long as the acute phase after transplant has passed (Orons et al., 1995a; Rostambeigi et al., 2013; Kodama et al., 2006).

Therefore, secondary considerations may be more important in making decisions about which treatment to use. For example, in the case of short concentric stenosis in a straight segment of the vessel, a simple PBA is a good option. Hamby et al. (Hamby et al., 2013) have suggested that tortuous or kinked vessels may benefit from stent placement. On the other hand, a tortuous vessel proximal to the stenosis may make stent advancement to the stenotic site difficult, and angioplasty would be a better option. Until now, there have been not prospective randomized studies to define specific criteria for the choice between stenting, PBA, or both.

Often, the decision has been made based on hepatic arterial anatomy and operator experience.

Our study suggests that PBA and stenting had similar long-term results (survival, graft function, and arterial patency).

In cadaveric liver transplantation, the previously reported balloon diameter for PBA has been indicated to be 3–6 mm (Kodama et al., 2006; Mondragon et al., 1994; Cotroneo et al., 2002; Raby et al., 1991). In our series, the balloon diameter was 4–5 mm.

The surgical management of HAS appears to be associated with greater complications in patients with liver transplantation and critical underlying disease. Whereas Abbasoglu (Abbasoglu et al., 1997) suggested a management trend leaning towards the operative approach, particularly for long stenosis, Saad et al. (2005) recommended that interventional treatment has comparable outcomes with higher safety and that surgical management might become complicated by hepatic artery thrombosis in 26% of cases (Saad et al., 2005).

Overall, reports show the success of interventional techniques to be between 70 and 100% (Abbasoglu et al., 1997; Orons et al., 1995b; Saad et al., 2005; Vignali et al., 2004), which is equal to or superior to the surgical approach. In our study, the rate of technical success was 100%.

The technical failures in HAS treatment are generally due to kinking or tortuosity of the hepatic artery and the inability of the balloon to pass beyond the stenotic site (Saad et al., 2005). Procedural complications occur in approximately 5–15% of cases (Orons et al., 1995b) and are mostly related to anastomosis and include hepatic arterial thrombosis, spasm, perforation, and dissection (Saad, 2007). In our study, the rate of complications was 5.8% (one case of dissection in group 1, immediately treated by stenting).

## Conclusions

This study suggests that PBA, stent, or both procedures show the same primary patency at 12 months.

It is probably not a definitive answer, but these treatments are safe and effective for extending graft survival in the context of graft shortages.

## Data Availability

The datasets used and/or analysed during the current study are available from the corresponding author on reasonable request.

## Abbreviations

GS: graft survival
PBA: percutaneaous balloon angioplasty
RI: resistive index
SAT: systolic acceleration time
HAS: hepatic artery stenosis
HAT: hepatic artery thrombosis

## References

[CR1] Abbasoglu O, Levy MF, Vodapally MS, Goldstein RM, Husberg BS, Gonwa TA et al (1997) Hepatic artery stenosis after liver transplantation-incidence, presentation, treatment, and long term outcome1. Transplantation 63(2) Disponible sur: https://journals.lww.com/transplantjournal/Fulltext/1997/01270/HEPATIC_ARTERY_STENOSIS_AFTER_LIVER.13.aspx 10.1097/00007890-199701270-00013 9020326

[CR2] Chen G-H, Wang G-Y, Yang Y, Li H, Lu M-Q, Cai C-J (2009). Single-center experience of therapeutic Management of Hepatic Artery Stenosis after Orthotopic liver transplantation. Eur Surg Res.

[CR3] Cotroneo AR, Di Stasi C, Cina A, De Gaetano AM, Evangelisti R, Paloni F (2002). Stent placement in four patients with hepatic artery stenosis or thrombosis after liver transplantation. J Vasc Interv Radiol.

[CR4] Dodd GD, Memel DS, Zajko AB, Baron RL, Santaguida LA (1994). Hepatic artery stenosis and thrombosis in transplant recipients: Doppler diagnosis with resistive index and systolic acceleration time. Radiology.

[CR5] Filippiadis DK, Binkert C, Pellerin O, Hoffmann RT, Krajina A, Pereira PL (2017). Cirse quality assurance document and standards for classification of complications: the Cirse classification system. Cardiovasc Intervent Radiol.

[CR6] Glockner JF, Forauer AR, Solomon H, Varma CR, Perman WH (2000). Three-dimensional gadolinium-enhanced MR angiography of vascular complications after liver transplantation. Am J Roentgenol.

[CR7] Goldsmith (1489). Complications after endovascular treatment of hepatic artery stenosis after liver transplantation. J Vasc Surg.

[CR8] Hamby BA (2013). Endovascular treatment of hepatic artery stenosis after liver transplantation. J Vasc Surg.

[CR9] Huang M, Shan H, Jiang Z, Li Z, Zhu K, Guan S (2006). The use of coronary stent in hepatic artery stenosis after orthotopic liver transplantation. Eur J Radiol.

[CR10] Kodama Y, Sakuhara Y, Abo D, Shimamura T, Furukawa H, Todo S (2006). Percutaneous transluminal angioplasty for hepatic artery stenosis after living donor liver transplantation. Liver Transpl.

[CR11] Mondragon RS, Karani JB, Heaton ND, Thomas S, Wong PY, O’Grady JG (1994). The use of percutaneous transluminal angioplasty in hepatic artery stenosis after transplantation. Transplantation.

[CR12] Mourad MM, Liossis C, Gunson BK (2014). Etiology and management of hepatic artery thrombosis after adult liver transplantation. Liver Transpl.

[CR13] Orons PD, Sheng R, Zajko AB (1995). Hepatic artery stenosis in liver transplant recipients: prevalence and cholangiographic appearance of associated biliary complications. Am J Roentgenol.

[CR14] Orons PD, Zajko AB, Bron KM, Trecha GT, Selby RR, Fung JJ (1995). Hepatic artery angioplasty after liver transplantation: experience in 21 allografts. J Vasc Interv Radiol.

[CR15] Raby N, Karani J, Thomas S, O’Grady J, Williams R (1991). Stenoses of vascular anastomoses after hepatic transplantation: treatment with balloon angioplasty. Am J Roentgenol.

[CR16] Rostambeigi N, Hunter D, Duval S, Chinnakotla S, Golzarian J (2013). Stent placement versus angioplasty for hepatic artery stenosis after liver transplant: a meta-analysis of case series. Eur Radiol.

[CR17] Saad WEA (2007). Management of Hepatic Artery Steno-Occlusive Complications after Liver Transplantation. Tech Vasc Interv Radiol.

[CR18] Saad WEA, Davies MG, Sahler L, Lee DE, Patel NC, Kitanosono T (2005). Hepatic artery stenosis in liver transplant recipients: primary treatment with percutaneous transluminal angioplasty. J Vasc Interv Radiol.

[CR19] Sabri SS, Saad WEA, Schmitt TM (2011). Endovascular therapy for hepatic artery stenosis and thrombosis following liver transplantation. Vasc Endovasc Surg.

[CR20] Ueno T, Jones G, Martin A, Ikegami T, Sanchez EQ, Chinnakotla S (2006). Clinical outcomes from hepatic artery stenting in liver transplantation. Liver Transpl.

[CR21] Vignali C, Bargellini I, Cioni R, Petruzzi P, Cicorelli A, Lazzereschi M (2004). Diagnosis and treatment of hepatic artery stenosis after orthotopic liver transplantation. Transplant Proc.

[CR22] Wozney P, Zajko A, Bron K, Point S, Starzl T (1986). Vascular complications after liver transplantation: a 5-year experience. Am J Roentgenol.

